# Heat as a Conductivity Factor of Electrically Conductive Yarns Woven into Fabric

**DOI:** 10.3390/ma15031202

**Published:** 2022-02-05

**Authors:** Željko Penava, Diana Šimić Penava, Željko Knezić

**Affiliations:** 1 Department of Textile Design and Management, Faculty of Textile Technology, University of Zagreb, 10000 Zagreb, Croatia; zeljko.knezic@ttf.unizg.hr; 2 Department of Engineering Mechanics, Faculty of Civil Engineering, University of Zagreb, 10000 Zagreb, Croatia; dianas@grad.unizg.hr

**Keywords:** woven fabric, plain weave, electrically conductive yarn resistance, heat, temperature

## Abstract

In recent years, more and more researchers have been focused on electrically conductive textiles that generate heat or transmit electrical signals and energy to embedded electrical components. In this paper, the dissipation of heat due to the flow of electric current at given voltages is investigated, and at the same time it is determined how this heat affects the change in the electrical resistance of the electrically conductive yarn in the immediate surroundings. Three fabric samples were woven in a plain weave with three types of different electrically conductive yarns. Three electrically conductive yarns are woven in parallel in the weft direction and separated from each other by one polyester (PES) yarn due to electrical insulaton. Conductive yarns are electrically connected so that the outer yarns are used for heating by the flow of electric current at a certain constant voltage, and the central yarn is used only to measure changes in electrical resistance. When electrothermally conductive fabrics are subjected to certain voltages over time, experimental results have shown that resistance values increase over a short period of time and then gradually decrease, while the temperature gradually increases and stabilizes over time. Based on the analysis of the obtained results of the ratio between the values of applied voltage and temperature to the electrically conductive yarns, the value of thermal dissipation in conductive yarns can be calculated in advance depending on the applied voltage. Furthermore, the obtained results can be further used in applications where conductive yarns are used as heaters for realistic prediction of the obtained heat.

## 1. Introduction

With the occurrence of smart textiles and smart clothing that collects information through built-in sensors, a new era in the production of textiles and clothing is in the making. One of the important properties of intelligent materials is certainly the ability to maintain body heat, as well as provide thermal protection. Materials that can perceive stimuli from the environment and adapt to them are also called electrically conductive textiles [1].

Electrically conductive yarns can also be used as electric heaters embedded directly as yarns into woven fabrics or knitted fabrics. Due to their ohmic resistance, electrically conductive yarns can develop heat by the flow of electric current. The heat can be regulated by adjusting the voltage value. This method of heating is most often used in medical clothing intended for patients, but also in sportswear for monitoring the fitness and physical condition of athletes during training [2,3,4,5].

The textile materials need to be enabled to have the necessary electrical properties for the transmission of electrical signals and energy and the possibility of installing sensors and electrical assemblies [6]. Therefore, they are partly converted into conductors of electric current, which is achieved by embedding the electrically conductive yarns [7]. Due to the influence of the environment, the electrical conductivity may decrease, i.e., the electrical resistance of electrically conductive yarns may increase. That is why it is very important to know their electrical characteristics. Many researchers have studied by experimental testing the influence of axial tensile load on changes in electrical resistance in woven conductive yarn [8,9]. Penava and associates studied the influence of woven fabric anisotropy and the influence of yarn count of electrically conductive yarns woven into fabric on the change in electrical resistance values. They have proved that, with the elongation of the electrically conductive fabric, the values of electrical resistance decrease with the increase in the yarn count value of the electrically conductive yarn [10].

The rapid development of science has made it possible to connect textile science with the electronics industry, which has resulted in the development of new practical textile products that are used in medicine, the military, etc. Today, the thermoelectric properties and resistances of electrically conductive yarns are increasingly being investigated. Temperature has a very important influence on the electrical properties of the electrically conductive yarns. Li Li and associates set up a mathematical equation by which they modeled the electrothermal characteristics of fabrics in the flow of electric current. The thermal conductivity coefficient was obtained by using the model [11]. An infrared photographic instrument is used to measure the temperature variation on the electrically conductive yarns through which an electric current of different power flows. High temperatures increase the electrical resistance of the yarn [12,13]. Thermal cameras are also used, which take thermal photos through a textile circuit in which the electrically conductive yarns are under different voltage values [14].

Thermal clothing in electro textiles occupies an increasingly important place. Poly-amide yarns coated with silver are most used for thermal clothing. Therefore, knowledge of its electromechanical and electrothermal properties is very important. The properties of electrically conductive yarns and the thermal balance of electrically conductive fabrics affect the calculation of the electrical resistance of electrically conductive fabrics. It is also important to research the heating fabrics that are increasingly used in the production of heating pillows and blankets. With the electrically conductive yarns that are embedded in heating fabrics, it is important to know what thermal effect is required, while maintaining the flexibility, comfort, and durability of the fabric. It is important to achieve the thermal control of conductive fabrics and this problem has been addressed by many researchers [15,16].

Tong and associates studied the impact of temperature change on the electrical resistance of textiles with embedded electrically conductive yarns because the temperature control of thermal textiles is important. Linear and contact resistance were studied experimentally. In their research, they have presented the quantitative relation between the electrical resistance of electrically conductive knitted fabric and temperature and proposed a theoretical model for controlling the temperature of electrically conductive knitted fabric in the wales and course direction. They concluded that the electrical resistance of any electrically conductive knitted fabric decreases significantly with increasing temperature [17,18]. The conductivity properties of the knitted fabric can be affected by variations in the structure of the knitted fabric. A certain geometrical model can provide a detailed mathematical description of a knitted fabric with the embedded electrically conductive yarns [19].

More recently, mesoscopic modeling has been used to investigate the thermal conductivity and thermal resistance of fabrics. Finite element methods have been used to present geometric models of fabric structures and to estimate its thermal properties [20].

Each type of electrically conductive yarns that is built into the knitted structure has its advantages and disadvantages. Experimental tests were performed, and the obtained results showed that steel yarn cannot be used for knitting, while polyamide yarn coated with silver is suitable for making knitted heating elements [21]. One of the new approaches to the study and research of in-put and operating parameters, and characteristics of yarn and knitted fabric that affect the thermal conductivity of elastic knitted fabrics is based on artificial intelligence. An original fuzzy-logic-based method was proposed [22].

A lack of information in the open literature about the maximum electrical current or voltage that can be applied on the conductive thread has been compensated by researchers in their paper, in which they investigate the ability of commercially available threads to handle and sustain electrical loads [23]. As can be seen from the review of the available literature, only the paper [18] investigated the influence of heat on the change of electrical characteristics of e-textiles, but it was a knitted structure made entirely of electrically conductive yarns in the function of the heating body (heating surface). As the behavior of electrically conductive yarns in the function of the heating element is different from the behavior of electrically conductive yarns in the function of conductor, the basic idea and goal of this experiment was to investigate heat dissipation due to electric current flow through electrically conductive yarn at a certain voltage. At the same time, it was observed how this heat affects the change in the resistance of the electrically conductive yarn as a function of the conductor located in the immediate surroundings. In practice, such a system is often encountered with woven or sewn electrically conductive yarns because the two outer yarns are used for power and are exposed to increased electric current, causing them to heat up, while the inner yarn serves as a signal line where electric currents are very small and do not affect heating yarns. However, the heat dissipation of the outer yarns acts directly on the central yarns and heats them up thus changing their electrical resistance as well. In this paper, the problem of heating the conductors used to power the built-in assemblies is primarily considered. Thus, the use of conductive yarns as a heating element is not studied, but rather we investigate heating as an undesirable phenomenon in the power supply of assemblies. What is the impact of and how much this change in electrical resistance affects the signal is an important information in the design of e-textiles.

It is important to emphasize that the influence of washing on heating and heat transfer from electrically conductive yarns is not investigated in this paper. As this is also an important factor in the design of e-textiles, it can certainly be the goal of some our further research.

## 2. Theoretical Analysis

Conductive yarn is the basic building element of e-textiles and is used to transmit electricity or electrical signals between electronic assemblies embedded into that e-textile. Electrically conductive yarn must therefore be characterized by low electrical resistance or good electrical conductivity, and the resistance to changes in these electrical quantities during mechanical action on the material itself. Such resistance is closely related to the structure of the electrically conductive yarns themselves, and the way they are embedded into textile material has a great influence. In addition to these, mechanical and structural factors, the electrical characteristics of the electrically conductive yarn are affected by changes in heat, whether it is an external source (body heat, heat sources, sun, infrared radiation, etc.) or heat caused by the flow of electrical current. With each electric conductor, the flow of electric current generates heat, which primarily depends on the resistance of that conductor. In the case of conductors made of copper, aluminum, silver, and gold, this is reduced to a minimum due to the very low electrical resistance of these materials. The electrically conductive yarns in their internal and surface structure consist of a network of conductive elements connected in series and in parallel, each of which can be represented as a resistor and, unlike conventional conductors, the generating heat by flowing electric current is significantly higher. In classic conductors, such heat generation (which consumes part of the electric current) is represented as thermal losses in the conductors.

Heat is also generated when an electrical current flows through the electrically conductive yarns embedded into the fabric. A certain amount of heat *Q* is generated in the fabric after a certain time *t* when the power *P* at a certain voltage *U* is applied to the conductive yarns embedded in the fabric. Therefore, the temperature will rise. The electrical resistance of such conductive yarn is denoted by *R*. The relationship between the heat, voltage and resistance over time is shown by Equation (1):

(1) $$Q=P\cdot t=\frac{U^{2}}{R}\cdot t$$

During the heating of the conductor, the heat is dissipated to the environment. This is called heat dissipation and is proportional to the product of the temperature variation and the dissipation coefficient of the conductor material *α* per unit time, Equation (2):

(2) $$\frac{Q_{d}}{t}=\frac{\alpha \cdot \left(T_{e}-T_{0}\right)}{t}$$

*T*_0_ is temperature of the environment, *T_e_* is final or equilibrium temperature, and *Q_d_* is the heat dissipation from the fabric to environment.

With the flow of the electric current, the temperature of the conductor continues to increase until the input power becomes equal to the amount of heat dissipation, when the conductor reaches a steady state. Then, heat dissipation can be related to resistance *R* and voltage *U* as follows (3):

(3) $$\alpha \cdot \left(T_{e}-T_{0}\right)=\frac{U^{2}}{R}$$

That is, the equilibrium temperature of the conductor can be calculated according to Equation (4):

(4) $$T_{e}=T_{0}+\frac{U^{2}}{\alpha \cdot R}$$

Since the change in the temperature of the conductor affects the change in its resistance, the dependence of the resistance *R* on the voltage *U* should also be included in Equation (3):

(5) R=R(U)

So, Equation (6) is obtained:

(6) $$T_{e}=T_{0}+\frac{U^{2}}{\alpha \cdot R\left(U\right)}$$

In this way, possible heat losses in the conductors can be predicted and calculated depending on the applied voltage, as well as changes in the resistance of the conductor when the equilibrium temperature is reached. If the resistance of the conductor changes significantly with the change of temperature, large deviations of the calculation itself are possible, and this is exactly the characteristic of the electrically conductive yarns. The dependence of the resistance of the conductive yarn on the temperature or the applied voltage will determine the level of accuracy of the equilibrium temperature prediction.

## 3. Experimental Procedure

### 3.1. Materials

In the experimental part of the paper, three different electrically conductive yarns were used (designations A, B, and C). The conductive yarns used for this paper were purchased from Shieldex Trading Inc. (Bremen, Germany) [24]. The electrically conductive yarns were silver-coated products that had antibacterial properties and were thermally and electrically conductive. All three electrically conductive yarns were made of high-strength polyamide (PA 6.6). The yarns are coated with 99% pure silver. The yarn with designation A had one ply and 17 filaments and its yarn count after coating with silver was 14.2 tex. Yarn count raw was 11.7 tex. Resistivity was about 250 Ω/m. The yarn with designation B was a two-plied yarn made from multifilament single yarns. It had 34 filaments and its yarn count after silver plating was 29.5 tex. Yarn count raw was 23.5 tex. Resistivity was about 200 Ω/m. The yarn with designation C was a two-plied yarn made from multifilament single yarns. It had 72 filaments and its yarn count after silver plating was 60.4 tex. Yarn count raw was 47 tex. Resistivity was about 55 Ω/m. The most important properties of electrically conductive yarns are shown in Table 1.

**Figure 1:**
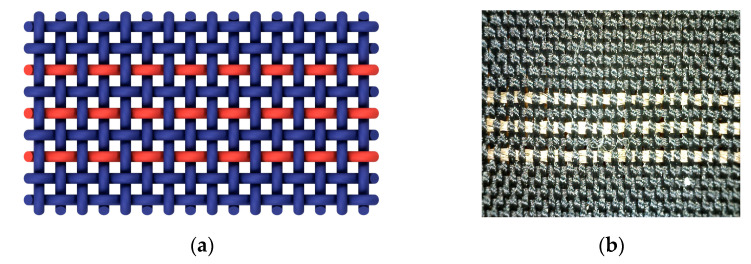
Plain weave: (**a**) woven fabric structure; (**b**) macro photo.

The tests were performed on fabric samples with structurally identical plain weave. The raw material composition of the warp and weft was PES. A warp yarn count and a weft yarn count were the same and amounted to 11 tex. Warp density was 56 cm^−1^, and weft density was 28 cm^−1^. The electrically conductive yarn, marked red, was woven in a weft according to Figure 1.

This design of woven conductive yarns was designed in the form of three parallel woven conductive yarns, separated from each other by one PES yarn (due to mutual electrical insulation). The fabric with electrically conductive yarn A was sample 1, the fabric with conductive yarn B was sample 2, and the fabric with electrically conductive yarn C was sample 3. All samples were made according to the authors’ design and based on the research plan and the production conditions and possibilities of the available loom.

### 3.2. Methods

Three parallel electrically conductive yarns were electrically connected in such way that the external yarns were used for heating by the flow of the electric current at a certain constant voltage, and the central yarn was used only to measure the change in electrical resistance. Thus, the central yarn (electrically separated on each side by one PES yarn) was a measuring yarn that was affected exclusively by thermal dissipation from the external yarns, which were heated by electrically conductive yarns. Three precision contactless sensors for measuring temperature at distance of 18.5 cm, based on the MLX90614 infrared temperature detector (Melexis, Ieper, Belgium), were installed above the entire system. The MLX90614 featured a built-in IR sensor signal processing to achieve thermometer accuracy and resolution.

Digitally processed and calculated object and environment temperatures were available with a resolution of 0.01 °C. They were available via a 2-wire serial SMBus connection and a compatible protocol (0.02 °C resolution) or via a 10-bit PWM (Pulse Width Modulated) output of the device. The MLX90614 was factory calibrated over a wide temperature range: −40 to 125 °C for environment temperature and −70 to 382.2 °C for object temperature. In order to minimize the measurement error, the mean value of temperature of the heated area measured with three contactless sensors was taken.

**Figure 2:**
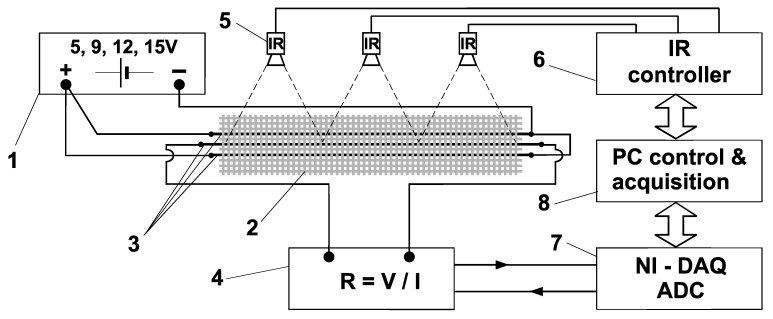
Schematic presentation of connecting sample and measuring system: 1—regulated power supply unit; 2—specimen of fabric; 3—conductive yarns; 4—resistance measuring circuit; 5—IR temperature sensors (MLX90614); 6—microcontroller for powering and acquisition temperature data; 7—NIDAQ converter; 8—personal computer for time control and data acquisition.

After the described preparation and setting of the measuring sample of fabric, the ex-ternal electrically conductive yarns were connected to the laboratory DC source with a constant voltage regulator, and the central woven conductive yarn was connected to the measuring system for measuring electrical resistance and via converter to PC. Contactless temperature sensors were placed above the test sample, which transmitted data to a PC via a microcontroller. All this is schematically shown in Figure 2.

For this specific research, the necessary algorithms were programmed on a PC, which was used for controlling the measurement process and collecting the measured data. In addition to measurement, the PC was used for the development of appropriate measurement and analytical software, as well as to complete the analysis of the measured data and the presentation of results.

### 3.3. Measurement Procedure

Before testing, the fabrics with the woven electrically conductive yarns were conditioned for 24 h under normal conditions: 65% humidity, at a temperature of 23 °C. The length of the sample of the tested fabric was 74 cm. This length was applied for practical reasons (production constraints, most commonly used in practice, as a compromise between the length-to-electricity ratio through all three types of samples). As is known from electrical engineering, the electrical resistance is in a linear relationship with the length of the conductor, which means that the results obtained are valid and can be applied to all lengths of electrically conductive yarns. On a specially prepared stand with insulated clamps, the fabric was preloaded with a force of 1 N, and the electrically conductive yarns were electrically connected to the connecting wires by mechanical joints. After placing the samples and connecting them to the connecting wires, the average temperature of the area with conductive yarns equal to the temperature of environment was measured. By switching on the constant voltage source of electric current to the external electrical yarns, their heating began, and thus the dissipation of heat to the central electrically conductive yarn. At the same time, the resistance of the central electrically conductive yarn and the average temperature of the heated area along the entire length of the tested fabric sample were measured. Values were measured and collected every second for 3600 s, to ensure that heat dissipation became stable and the temperature of the measured area constant. After 3600 s, the electric current source was turned off, the sample was left to cool to ambient temperature, and the measurement was repeated with the following constant voltage. Constant voltages of 5 V, 9 V, 12 V and 15 V were used. All measured resistance and temperature data were stored on a PC and prepared for processing after the completion of all sample tests. Five measurements were performed for each voltage value, which means that twenty samples were tested for each yarn. That is a total of 60 tests for all 3 yarns.

## 4. Results and Discussion

**Figure 3:**
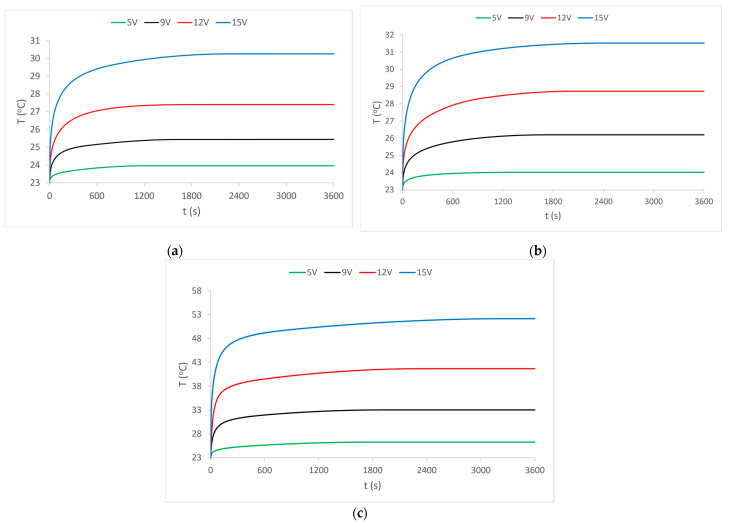
Temperature movement of the electrically conductive part of the sample at certain voltages: (**a**) sample 1; (**b**) sample 2; (**c**) sample 3.

As this research is based on heat and heat dissipation, the results of measuring the temperature of the heated area on a fabric sample with the electrically conductive yarns at supply voltages of 5 V, 9 V, 12 V and 15 V are first presented. The diagrams show the mean or average values of the obtained measurements. For sample 1, the results are shown in Figure 3a, for sample 2 in Figure 3b, and for sample 3 in Figure 3c.

The results of temperature measurements are shown by the diagram of heat development over time. The diagram for sample 1, Figure 3a shows a rapid increase in temperature in the first minute of the heating process, and a slower increase in temperature in the next 10 min of the process. It can be observed that the temperature reaches its constant values only after 20 min of the heating process.

The diagram also shows the influence of the supply voltage because a separate temperature rise curve is shown for each applied voltage. As expected, the lowest voltage (5V) gives the smallest increase in temperature as well as the lowest and fastest constant temperature is reached. The highest voltage (15 V) causes a strong and rapid initial rise in temperature as well as the highest temperature, but to achieve constant temperature values takes significantly longer, even 30 min.

The results of temperature measurements in sample 2 and sample 3 (in Figure 3b,c) show similar values and almost identical behavior, except those of sample 3, where more temperature values were developed at all voltage values. It is also noticeable that it takes a longer period for the temperature to reach its constant value.

**Figure 4:**
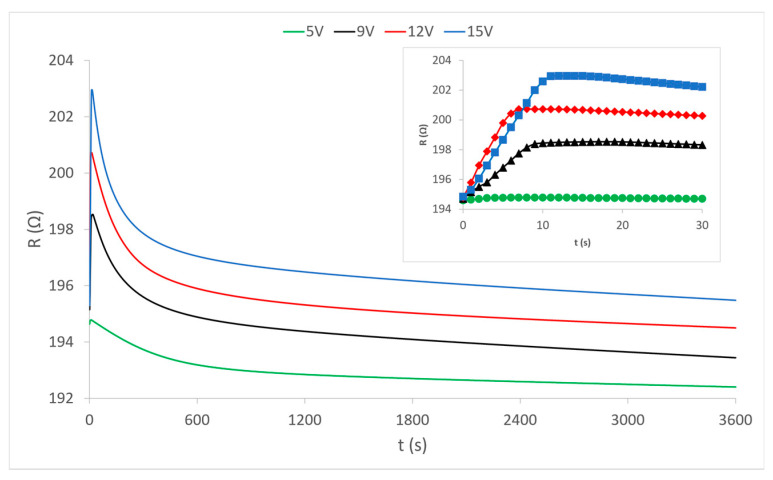
Change in the electrical resistance of the electrically conductive yarn of sample 1 under the influence of temperature change at given voltages.

**Figure 5:**
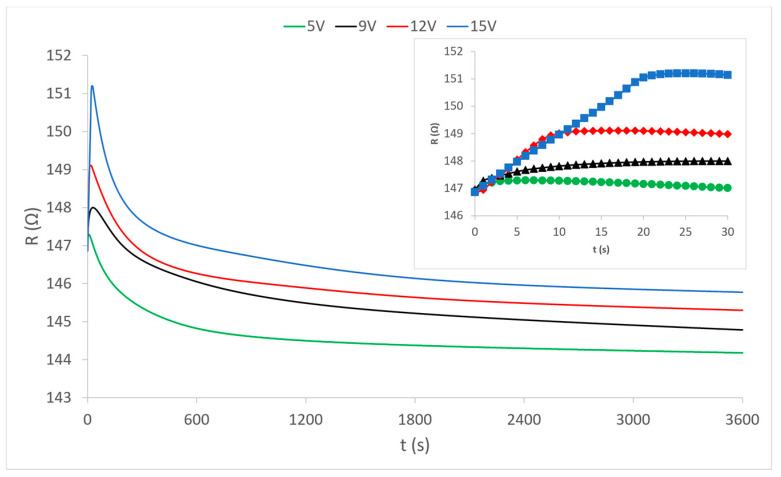
Change in resistance of the electrically conductive yarn of sample 2 under the influence of heat at given voltages.

**Figure 6:**
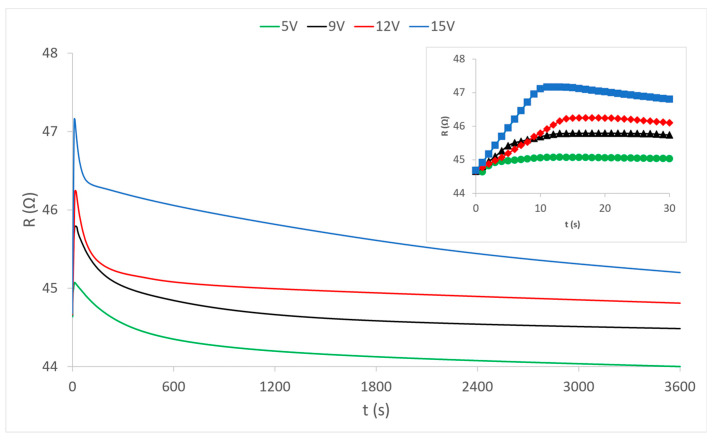
Change in the electrical resistance of the electrically conductive yarn of sample 3 under the influence of heat by heating at given voltage values.

Having determined how the heating process develops by the flow of electric current through the external electrically conductive yarns (Figure 3), it can be considered that the heat dissipation of these yarns affects the central yarn. Heat is transferred to the central yarn indirectly by contact (via the connecting yarns of the PES warp and weft) and directly by radiation into the environment. It is already known that the increase in temperature is the main factor responsible for the change in the electrical resistance of electrically conductive yarns. The electrical resistance whose change under the influence of heat is shown in the diagrams in Figure 4, Figure 5 and Figure 6 was measured.

The diagram in Figure 4 shows the measured continuous changes in the resistance value in the central electrically conductive yarn under the influence of the heating of the outer electrically conductive yarns at different voltages. The change in resistance occurs exclusively by the action of heat because the central yarn is completely electrically isolated from electrical sources and electric power flow in external electrically conductive yarns. The presented curves show that the resistance at the very beginning (first 10 s) of the heating process increases sharply, after which it gradually decreases in the first 10 min of heating. Abrupt changes in the first ten seconds of the process are shown magnified on the separated partial diagram in the upper right corner of the diagram view. In the continuation of the process, the resistance values very slowly continue to fall asymptotically approaching the values before the action of heat, and even below them. Each curve separately shows changes in the values of electrical resistance at applied voltage values (5 V, 9 V, 12 V and 15 V) at which the external electrically conductive yarns are heated. There is an interesting phenomenon that at the weakest heating (5 V) the increase in resistance is almost imperceptible at the beginning, and during the further process of heat action, the electrical resistance falls slightly below its nominal value before heating. It can be concluded that the heating power at a voltage of 5V is too low to ensure sufficient heat dissipation from the heated external yarns. With stronger heating (higher voltages) the electrical resistance does not decrease but becomes higher than its nominal value. Thus, increased heating causes an increase in the electrical resistance of the conductive yarn. However, this phenomenon is not the rule because different electrically conductive yarns behave differently, as can be seen in the diagram of sample 2 (Figure 5).

The presented results of measuring the electrical resistance of sample 2 (Figure 5) show a very similar movement of the electrical resistance of the electrically conductive yarn under the influence of the generated heat at a given four voltage values. In the same way, there is a sharp increase in the first 20 s of the process, then a decrease in value over the next 10 min and then a further slight decrease. However, unlike sample 1, here the decrease extends significantly below the value of the electrical resistance of the yarn before heating, so it can be said that heating ultimately reduces the electrical resistance of the conductive yarn. This is a paradox in the electrotechnical sense because it is rarely found in classical conductors and electrotechnical elements (except for special elements of the NTC resistor type, which are specially made as resistors with a negative temperature coefficient). In textiles, i.e., in the electrically conductive yarns, this can be explained by the influence of yarn structure (plied yarn) where under the influence of heat there is most likely microscopic thermal expansion of yarn material, and thus better and more quality contact between the plied yarns, resulting in reduced overall electrical resistance. In this sample (sample 2), it is necessary to notice evident deviations in the increase and decrease in the resistance value by heating at different voltage values. As there are no such deviations in sample 1, it can be concluded that the structure of yarn plays a significant role here also. This is confirmed in many elements in the presentation of the results of sample 3 in the following diagram (Figure 6).

The results of measuring the change in the electrical resistance of the electrically conductive yarn of sample 3 under the influence of heat at given heating voltage conditions are shown in the diagram in Figure 6. The electrically conductive yarn in this sample is characterized by low nominal electrical resistance (44.5 Ω), and therefore by significantly higher electric current and consequently the higher heating power at all given voltage values (5 V, 9 V, 12 V and 15 V). This higher power is of course reflected in the increased heat dissipation of the external electrically conductive yarns and faster and stronger heating of the central electrically conductive yarn. This can be seen in the extremely rapid initial increase in electrical resistance, but also in its rapid decline. These initial transients are clearly visible in the separated partial diagram in the upper right corner of the diagram view. In this sample, it is interesting to note that, despite its plied structure, it behaves more similarly to the first sample with a conductive one ply yarn.

This primarily refers to the values of electrical resistance, which do not fall below the nominal value during prolonged heating, and this affects the increase in the total resistance values of the electrically conductive yarn with increasing temperature. The exception is heating at the lowest voltage (and thus the lowest power) of 5 V. Here, the values of electrical resistance fall below the nominal value, as is the case with sample 2. The reasons for such values are most likely to be sought again in the structure of the electrically conductive yarn. In this sample, at the strongest heating (15 V), the curve of electrical resistance measurement should be pointed out, where the values of decreasing electrical resistance did not stabilize until the end of the observed time (1 h in total). It can be assumed that the heat dissipation in this case was too great and the heating of the outer yarns was excessive (too high current due to low resistance of the conductive yarn results in high electrical power, which can lead to the structural deformation of heated yarns). From this follows the conclusion that in the transmission of electricity through the electrically conductive yarns of low electrical resistance, an electronic limit of current, i.e., total power, must be used.

**Figure 7:**
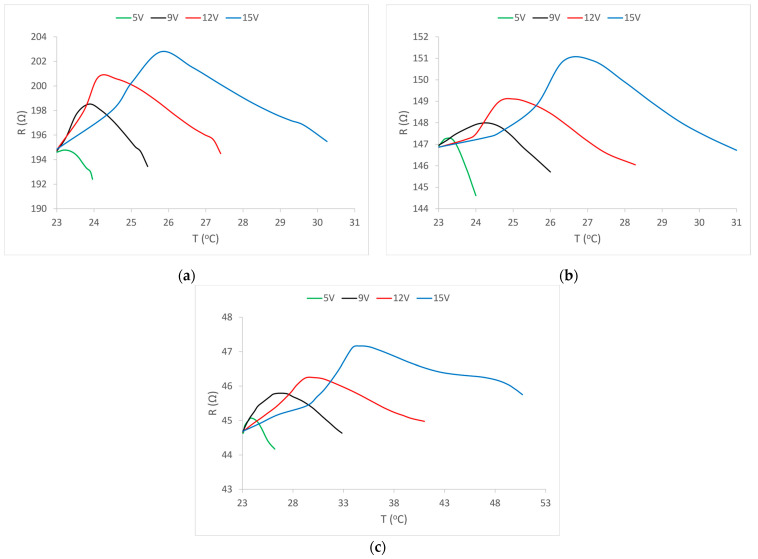
Change in the electrical resistance of the electrically conductive yarn with increasing temperatures at certain voltage values: (**a**) sample 1; (**b**) sample 2; (**c**) sample 3. In the continuation of the presentation of the results, diagrams of the change in resistance depending on the increase in the temperature of the conductive yarn are given.

The temperature changes of the electrical resistance values of the electrically conductive yarn in sample 2 are shown in Figure 7b. As with sample 1, the initial increase in value is visible, followed by a decrease below the nominal (initial) value of electrical resistance. In contrast to sample 1, in sample 2, the increase in the resistance value takes place within a larger temperature range for all values of applied voltages. After reaching the maximum, there is a decrease in resistance value almost identical to that of sample 1.

Figure 7a shows the change in the electrical resistance of the electrically conductive yarn during the increase in temperature for sample 1 at all four applied voltages. As with the resistance change diagram over time, a sharp increase in the resistance value is observed with even a very small increase in temperature, followed by a marked decrease in the resistance value. The smallest and slowest increase in the resistance value is at the lowest voltage or at the lowest energy flow. At all voltages, after the increase, there is a decrease in the value of resistance that falls below the nominal (initial) value. The change is most pronounced at the highest voltage, i.e., at the highest flow of energy through the electrically conductive yarn.

For sample 3, the change in electrical resistance of the electrically conductive yarn due to the action of temperature is shown in Figure 7c. In this sample, a sharp increase in the resistance value is noticeable even at the lowest voltage. The electrical resistance of electrically conductive yarns in this sample behaves somewhat differently from the resistance of yarns in sample 1 and sample 2, but here, too, the initial increase in resistance is visible at all applied voltages, followed by a decrease. In this sample, changes in electrical resistance (increase, maximum and decrease in value) occur in a much larger temperature range. This can be explained by the lowest nominal electrical resistance, which therefore allows the highest flow of electric current under all voltage conditions and thus the highest power consumed in the flow to heat the conductor. In this sample, the largest deviation is at the highest voltage and thus the highest energy flow, which therefore causes the largest increase in temperature. Such an increase in temperature can have a negative effect on structural changes in the electrically conductive filament. Therefore, we have already mentioned that, in the transmission of electricity through electrically conductive threads of low electrical resistance, an electronic limit of current must be used, and thus the total power.

In conclusion, the diagrams (Figure 7a–c) show that temperature plays a significant role in changing the electrical resistance of electrically conductive yarns.

In all tested samples, three electrically conductive yarns were woven in the weft direction. The central yarn was used to measure changes in resistance, and the outer yarns for heating by the flow of electricity under given voltage conditions. As these outer yarns were identical to the central yarn in all elements (yarn type, yarn count, length tests, yarn weaving, and nominal electrical resistance), it is assumed that the measured changes in the electrical resistance value of the central electrically conductive yarn may apply to these outer yarns. This is the case especially because the electrical resistance in the outer yarns was measured before the voltage was switched on and after the heating voltage was off, and the results were identical to the electrical resistance measured on the central yarn. The following diagrams show how, depending on the selected voltage value, heat developed in a particular sample.

**Figure 8:**
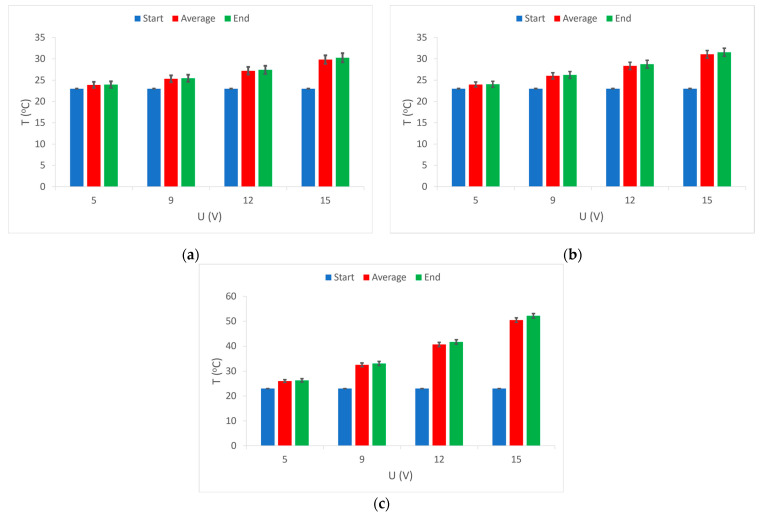
Temperature measurement results at selected voltage values: (**a**) sample 1; (**b**) sample 2; (**c**) sample 3.

Figure 8 shows the results of measuring the initial, final, and average temperature of the measuring area depending on the supply voltage. As can be seen from the diagram, for each sample at all voltages the initial temperature was the same, which is understandable since the measurements took place in the environment under standard conditions of 20 °C and 65% humidity. The diagrams for all samples show that, at higher voltages (and thus higher electrical power), the temperature in the electrically conductive yarns had a larger increase. Almost identical values of the average and final temperature indicate a quick start of the process of reaching a continuous temperature and thus stable heat dissipation. The results of temperature measurements in sample 1 and sample 2 show almost no differences between them with all four applied voltages (Figure 8a,b). This is a consequence of the larger electrical resistance of the applied conductive yarns (194.6 Ω and 146.8 Ω) as well as their similarity (the only difference is that in the first sample it is a single-yarn and, in the second, it is a plied yarn of the same fineness and composition). In sample 3 (Figure 8c), a difference is noticeable compared to the previous samples, because the temperature in this case reaches significantly higher values at all selected voltage values. This temperature development can be explained by the relatively low nominal electrical resistance (44.5 Ω) of the applied conductive yarn. With the same voltage and such a low electrical resistance, a higher electric current occurs, resulting in a significantly higher electrical power, the part of which is converted into heat.

Furthermore, it is certainly interesting to show comparative values of the electrical resistance of the conductive yarns in the samples, in relation to the applied voltage.

**Figure 9:**
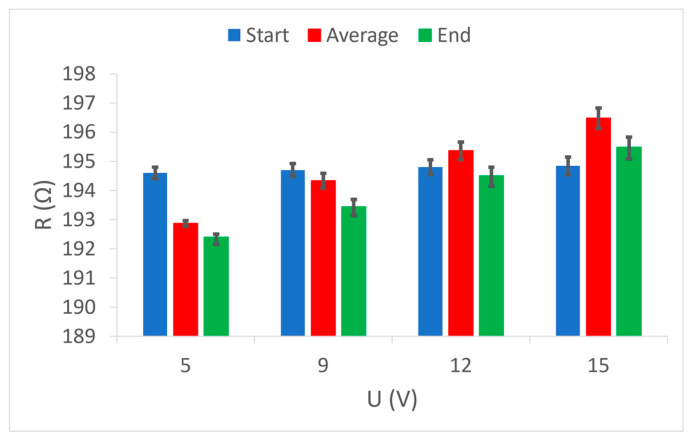
Comparison of three resistance values (start, average, end) at given voltage values in sample 1.

Figure 9 shows the initial, final, and average values of the electrical resistance of the electrically conductive yarns in sample 1, grouped according to the voltages used. It is cleared that the initial resistance values are always the same because the measurement always started under the same conditions. The final resistance values show how the heat affected the value of the electrical resistance of the conductive yarns in the sample. There is a noticeable reduction in the final resistance at lower voltages (5 V, 9 V and 12 V), while at the highest voltage (15 V) this resistance is higher than the initial one. When the highest electrical power applies on the electrically conductive yarn at the highest voltage, the heating is the highest, and this is a factor that affects the increase in electrical resistance in all conventional electrical conductors. The results of the average values of electrical resistance during the entire duration of the measurements show a noticeably higher value than the final values. This indicates a relatively slow calming of the initial jumping reactions to the action of heat and the gradual achievement of a continuous value of the electrically conductive yarn resistance.

The test results show that electrically conductive yarns have a kind of anomaly in relation to conventional electrical conductors. At conductive yarns, low heating reduces the values of electrical resistance, but increased heating causes an increase in electrical resistance. Therefore, in the use of electrically conductive yarns as power conductors, it is always necessary to limit the transmission of electric current to a level at which the increase in electrical resistance has not yet occurred.

**Figure 10:**
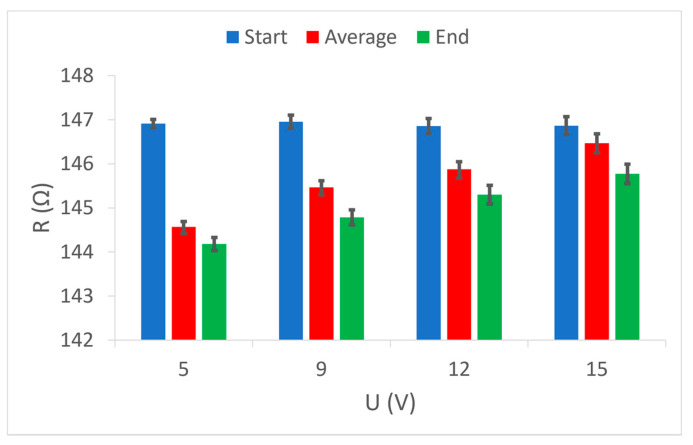
Comparison of three resistance values (start, average, end) at given voltage values in sample 2.

Comparative measurements results of electrical resistance at given voltage values (5 V, 9 V, 12 V and 15 V) in sample 2 show equal initial values of electrical resistance (Figure 10). This sample should indicate the results of the final values which show a decrease in electrical resistance at all voltages used. Here, it is noticeable that this decrease is most strongly at the lowest voltage (5 V), i.e., at the weakest heating, and increases almost linearly with the increasing voltage, i.e., increasing heating. In sample 2, despite the lower electrical resistance of electrically conductive yarns compared to sample 1, even at the highest applied voltage (hence the highest electrical power), there is no increase in electrical resistance above the nominal (initial). The difference between the electrically conductive yarns in sample 1 and in sample 2 is that in sample 1 the yarn is one ply and in sample 2 is a two-plied yarn, but of the same fineness and composition. It is easy to conclude that the electrically conductive yarn of the same characteristics in the plied form has significantly better electrical characteristics than the one ply yarn, and it is certainly capable of better transmission of higher levels of electrical power. In sample 2, it should be noted that at all voltages the difference between the results of the average and the final values is almost the same. This indicates a uniform thermal reaction of the electrically conductive yarn at all supply voltages, i.e., uniform calming of the initial oscillations and relatively fast achievement of a continuous value of electrical resistance. This element of uniformity is another indicator of better electrical power transmission in plied electrically conductive yarn.

**Figure 11:**
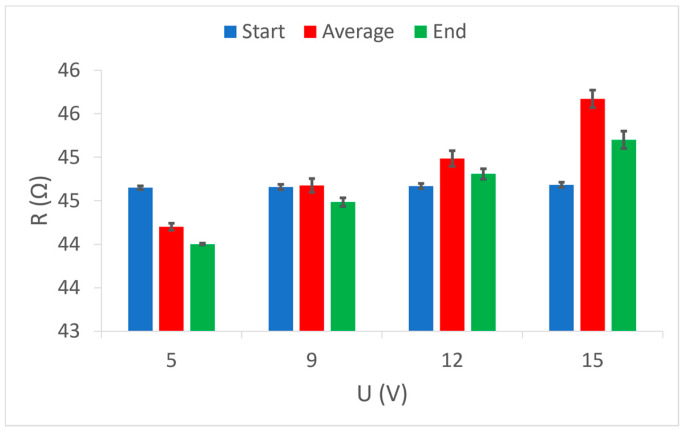
Comparison of three resistance values (start, average, and end) at given voltage values in sample 3.

Electrically conductive yarns woven into sample 3 differ significantly from the electrically conductive yarns in sample 1 and sample 2. The difference is in the nominal electrical resistance, yarn fineness, cross-section, weave, and raw material composition. The similarity is only with sample 2 because in sample 3, the yarns are also two-plied. In Figure 11, the process of resistance change under the influence of heat is visible, which are similarly to sample 1 where a one-ply electrically conductive yarn was used. At low voltages (5 V and 9 V), and thus low transmission power, the electrical resistance decreases, while at higher voltages and electric current flows, the electrical resistance of electrically conductive yarns increases. It is good to note that at the highest voltage (15 V) and consequently the highest heating, the average measurement value of electrical resistance results in deviation at the final value, which indicates excessive heat dissipation and inability of electrically conductive yarns to achieve a continuous electrical resistance value in the observed period. This once again confirms the necessity of installing a current limiter, and thus electrical power through electrically conductive yarn. Such installation controls the electric current level and, consequently, the development of excessive heat dissipation.

**Figure 12:**
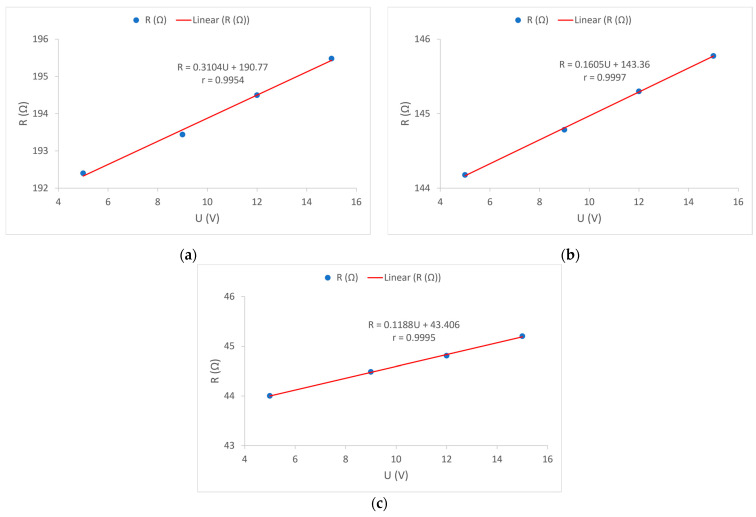
Dependence of electrical resistance *R* of electrically conductive yarns on supply voltage *U*: (**a**) Sample 1; (**b**) Sample 2; (**c**) Sample 3.

In the further interpretation of the results, the mutual relation and dependence of electrical resistance and voltage in each of the samples were observed and shown by correlation diagrams, Figure 12.

Figure 12 shows the correlation between the electrical resistance of electrically conductive yarns *R* and the values of supply voltage *U* in all tested samples. To determine this dependence, the results of the average electrical resistance at a given voltage were used. A very high correlation coefficient *r* was obtained, and the highest is for sample 2, which amounts to r = 0.9997. Such high correlation coefficients in all samples indicate a high linearity of the relationship between the electrical resistance of electrically conductive yarns and the applied voltages. From the attached graphs, it can also be seen that the dependence of the change in resistance on the applied voltage is lower if the nominal electrical resistance of the electrically conductive yarns is lower, Figure 12.

It remains to be seen how the supply voltage value affects the process of heat development in the electrically conductive yarns, i.e., the dependence of temperature on the applied voltage.

For sample 1, the function *R(U)* is:

(7) R=0.3104·U+190.77

**Figure 13:**
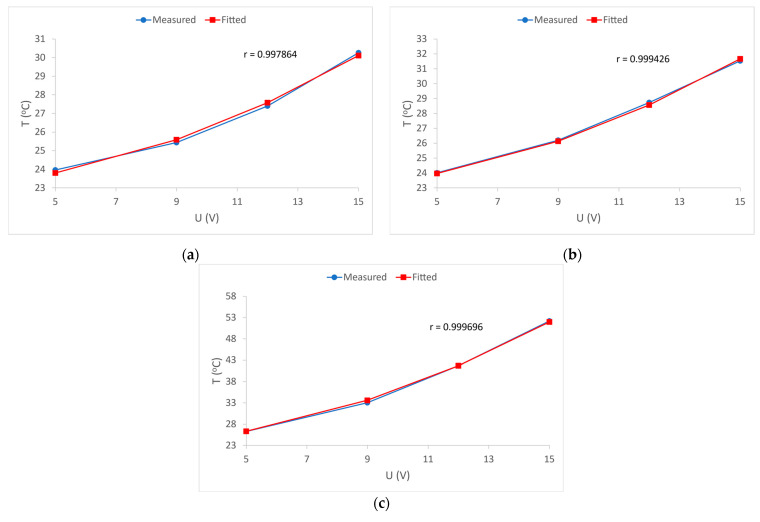
Dependence of equilibrium temperature *T_e_* of electrically conductive yarns on supply voltage *U*: (**a**) sample 1; (**b**) sample 2; (**c**) sample 3.

By substituting Equation (7) into Equation (6), Equation (8) is obtained, which is used to fit the measured *T*–*U* curve of sample 1 shown in Figure 13a.

(8) $$T_{e}=23+\frac{U^{2}}{\alpha \cdot \left(0.3104\cdot U+190.77\right)}$$

Figure 13 shows the curves as a graphical representation of the measured average temperatures *T* as well as their fitted shape, both as functions of the supply voltage *U*. The linear dependence of the resistance on the supply voltage has been previously shown and proven (Figure 12). Using these results and with the measured values of initial and final temperature, the heat dissipation coefficient for the electrically conductive yarns in each sample was calculated based on Equation (6). With this information, and with the inclusion of the dependence *R(U)* in Equation (6), the theoretical dependence of thermal dissipation depending on the applied voltage was obtained. The obtained correlation coefficients *r* for all samples shows a very high dependence of temperature *T* on the applied voltage *U*, Figure 12. For sample 1, the dissipation coefficient is *α* = 0.1619 (W/°C); for sample 2 the dissipation coefficient is *α* = 0.1782 (W/°C), and for sample 3 the dissipation coefficient is *α* = 0.1718 (W/°C). For all three samples the heat dissipation coefficients are approximately equal and very high correlation coefficients are obtained (Figure 13). This means that Equation (8) can be used to predict voltage-dependent temperature, for sample 1. By including the dissipation coefficient *α* for sample 1 in Equation (8), the following final Equation is obtained:

(9) $$T_{e}=23+\frac{U^{2}}{0.1619\cdot \left(0.3104\cdot U+190.77\right)}$$

Equation (9) can be used to predict the equilibrium temperature in the fabrics with the electrically conductive yarns intended to transmit power supply.

With this procedure, the amount of thermal dissipation depending on the voltage in electrically conductive yarns can be calculated in advance. It is also possible to predict changes in the electrical resistance of an electrically conductive yarn when a stable temperature is reached. Of course, if the electrical resistance of an electrically conductive yarn under the influence of heat shows significant oscillations, there may be a significant deviation in the calculation itself. This kind of knowledge leads to the conclusion that the level of accuracy of the calculation of stable thermal dissipation of electrically conductive yarn as well as changes in its resistance under the influence of heat strongly depend on the structure, fineness, material, and nominal electrical resistance of electrically conductive yarn.

## 5. Conclusions

Apart from mechanical influences, the action of heat plays a significant role in changes in the electrical resistance of conductive yarns. In addition to external heat sources, electrically conductive yarns are also affected by the heat generated by the flow of electric current through them. The increase in temperature in the conductive yarns, whether from an external source or due to the flow of electric current, results in a linear increase in the resistance of the conductive yarns in the fabric. The dependence of the change in electrical resistance on the applied supply voltage is lower if the nominal electrical resistance of electrically conductive yarns is lower.

Plied conductive yarn shows better characteristics for electricity transmission because it develops a uniform thermal reaction at different voltages, i.e., uniform calming of initial oscillations and relatively fast achievement of a continuous value of electrical resistance.

The analysis of the relationship between the applied voltage and the temperature on the conductive yarns can be further used to practically predict the influence of heat on the electrical resistance of the conductive yarns. This relationship can also be used in applications where conductive yarns are used as heaters to realistically predict the obtained heat.

The level of accuracy of the calculation of the stable thermal dissipation of electrically conductive yarn, as well as changes in its resistance under the influence of heat, depend on the structure, fineness and material, and the nominal electrical resistance of electrically conductive yarn.

In general, the total resistance of the conductive yarns in the fabric changes significantly when the fabric is heated. Consequently, it is necessary to pay more attention to this characteristic of electrically conductive yarns when designing e-textiles.

## Figures and Tables

**Table 1:** Characteristic parameters of the electroconductive yarns.

Code Name	A	B	C
Yarn count raw (tex)	11.7	23.5	47.0
Yarn count silverized (tex)	14.2	29.5	60.4
Resistivity	250 Ω/m	200 Ω/m	55 Ω/m

## Data Availability

Not applicable.

## References

[1] Tao X. (2001). Smart technology for textiles and clothing—Introduction and overview. Smart Fibres, Fabrics and Clothing.

[2] Fang Y., Chen G., Bick M., Chen J. (2021). Smart textiles for personalized thermoregulation. Chem. Soc. Rev..

[3] Xue P., Tao X., Leung M.Y., Zhang H. (2005). Electromechanical properties of conductive fibres, yarns and fabrics. Wearable Electron. Photonics.

[4] Cherenack K., Van Pieterson L. (2012). Smart textiles: Challenges and opportunities. J. Appl. Phys..

[5] Repon M.R., Laureckiene G., Mikucioniene D. (2021). The Influence of Electro-Conductive Compression Knits Wearing Conditions on Heating Characteristics. Materials.

[6] Cochrane C., Koncar V., Lewandowski M., Dufour C. (2007). Design and Development of a Flexible Strain Sensor for Textile Structures Based on a Conductive Polymer Composite. Sensors.

[7] Maity S., Chatterjee A. (2014). Polypyrrole Based Electro-Conductive Cotton Yarn. Int. J. Text. Sci..

[8] Zhang H., Tao X., Wang S., Yu T. (2005). Electro-Mechanical Properties of Knitted Fabric Made from Conductive Multi-Filament Yarn Under Unidirectional Extension. Text. Res. J..

[9] Ryu J.W., Hong J.K., Kim H.J., Jee Y.J., Kwon S.Y., Yoon N.S. (2010). Effect of Strain Change of Electrically Conductive Yarn on Electric Resistance and Its Theoretical Analysis. Sen-I Gakkaishi.

[10] Knezić Ž., Penava Ž., Šimić Penava D., Rogale D. (2021). The Impact of Elongation on Change in Electrical Resistance of Electrically Conductive Yarns Woven into Fabric. Materials.

[11] Li L., Au W., Ding F., Hua T., Wong K.S. (2014). Wearable electronic design: Electrothermal properties of conductive knitted fabrics. Text. Res. J..

[12] Li Y., Liu H., Li X. (2017). Thermal-electrical properties and resistance stability of silver coated yarns. AIP Conf. Proc..

[13] Bhat N.V., Seshadri D.T., Nate M.M., Gore A.V. (2006). Development of Conductive Cotton Fabrics for Heating Devices. J. Appl. Polym. Sci..

[14] Sezgin H., Bahadir S.K., Boke Y.E., Kalaoglu F. Effect of Different Conductive Yarns on Heating Behaviour of Fabrics. Proceedings of the 4th Annual International Conference on Textiles and Fashion.

[15] Tong J., Li L., Tao X. (2015). Thermal Regulation of Electrically Conducting Fabrics. Handbook of Smart Textiles.

[16] Shahzad A., Jabbar A., Irfan M., Qadir M.B., Ahmad Z. (2020). Electrical resistive heating characterization of conductive hybrid staple spun yarns. Text. Inst..

[17] Tong J., Liu S., Yang C., Li L. (2015). Modeling of package-free flexible conductive fabric with thermal regulation where temperature can be customized. Text. Res. J..

[18] Tong J., Ding F., Tao X., Au W.M., Li L. (2014). Temperature effect on the conductivity of knitted fabrics embedded with conducting yarns. Text. Res. J..

[19] Liu S., Yang C., Zhao Y., Tao X., Tong J., Li L. (2016). The impact of float stitches on the resistance of conductive knitted structures. Text. Res. J..

[20] Siddiqui M., Sun D. (2018). Thermal Analysis of Conventional and Performance Plain Woven Fabrics by Finite Element Method. J. Ind. Text..

[21] Šahta I., Baltina I., Truskovska N., Blums J., Deksnis E. (2014). Selection of conductive yarns for knitting an electrical heating element. WIT Trans. Built Environ..

[22] Fayala F., Alibi H., Jemni A., Zeng X. (2014). Study the Effect of Operating Parameters and Intrinsic Features of Yarn and Fabric on Thermal Conductivity of Stretch Knitted Fabrics Using Artificial Intelligence System. Fibers Polym..

[23] Stavrakis A.K., Simic M., Stojanović G.M. (2021). Electrical Characterization of Conductive Threads for Textile Electronics. Electronics.

[24] Shieldex Trading, Inc. “Yarns/Threads”, Shieldex Trading, Inc. 1 January 2018. https://www.shieldextrading.net/products/yarns-threads/.

